# Ablation of Myeloid Cell MRP8 Ameliorates Nephrotoxic Serum-induced Glomerulonephritis by Affecting Macrophage Characterization through Intraglomerular Crosstalk

**DOI:** 10.1038/s41598-020-59970-9

**Published:** 2020-02-20

**Authors:** Yusuke Hata, Takashige Kuwabara, Kiyoshi Mori, Youngna Kan, Yuki Sato, Shuro Umemoto, Daisuke Fujimoto, Tomoko Kanki, Yoshihiko Nishiguchi, Hideki Yokoi, Yutaka Kakizoe, Yuichiro Izumi, Motoko Yanagita, Masashi Mukoyama

**Affiliations:** 1 Present Address: Department of Nephrology, Kumamoto University Graduate School of Medical Sciences, Kumamoto, Japan; 2 Department of Nephrology, Kyoto University Graduate School of Medicine, Kyoto, Japan; 3 Present Address: Department of Nephrology and Kidney Research, Shizuoka General Hospital, Shizuoka, Japan; 4 Present Address: School of Pharmaceutical Sciences, University of Shizuoka, Shizuoka, Japan; 5 Institute for the Advanced Study of Human Biology (ASHBi), Kyoto University, Kyoto, Japan

**Keywords:** Diseases, Nephrology

## Abstract

Toll-like receptor 4 (TLR4) and one of its endogenous ligands myeloid-related protein 8 (MRP8 or S100A8), especially expressed in macrophages, play an important role in diabetic nephropathy and autoimmune disorders. However, detailed mechanisms and consequence of MRP8 expression remain unknown, partly due to embryonic lethality of MRP8 knockout mice. In this study, Myeloid lineage cell-specific MRP8 knockout mice were generated, and nephrotoxic serum-induced glomerulonephritis was developed. Mice with conditional ablation of MRP8 gene in myeloid cells exhibited less severe histological damage, proteinuria and inflammatory changes compared to control mice. Mechanism of MRP8 upregulation was investigated using cultured cells. Co-culture of macrophages with mesangial cells or mesangial cell-conditioned media, but not with proximal tubules, markedly upregulated MRP8 gene expression and inflammatory M1 phenotype in macrophages, which was attenuated in MRP8-deleted bone marrow-derived macrophages. Effects of MRP8 deletion was further studied in the context of macrophage-inducible C-type lectin (Mincle), which is critically involved in maintenance of M1 phenotype of macrophages. MRP8 ablation in myeloid cells suppressed the induction of Mincle expression on macrophages in glomerulonephritis. Thus, we propose that intraglomerular crosstalk between mesangial cells and macrophages plays a role in inflammatory changes in glomerulonephritis, and MRP8-dependent Mincle expression in macrophage may be involved in the process.

## Introduction

The prevalence of chronic kidney disease requiring renal replacement therapy is increasing sharply all over the world^1^. Glomerulonephritis is among the second most prevalent causes of end-stage renal disease. Especially, crescentic glomerulonephritis is a still unresolved disease, in which renal prognosis is rapidly declining unless managed properly using glucocorticoids or immunosuppressants^2^. Such situation prompted us to explore the novel therapeutic strategies for crescentic glomerular diseases.

The immune system has originally emerged as a defense mechanism against non-self antigens. In these days, accumulating evidence has shown that the system is also involved in the pathogenesis of various non-communicable diseases, including diabetes, obesity, cancer, and autoimmune disease even without infection^3,4^. Such sterile inflammation is referred to as “chronic inflammation” caused by interactions between pattern recognition receptors such as toll-like receptors and their endogenous ligands, collectively called damage-associated molecular patterns (DAMPs)^5^. Although the precise role for chronic inflammation has yet to be clarified in kidney disease, we and others recently revealed the pathogenic role of toll-like receptor 4 (TLR4) in the development and progression of diabetic nephropathy^6–8^ and crescentic glomerulonephritis^9,10^. With regard to DAMPs, Myeloid-related protein 8 (MRP8, also known as S100A8 or calgranulin A) was originally identified as a cytoplasmic calcium-binding protein in neutrophils and monocytes^11^. Several key reports have shown that MRP8, in association with its family member MRP14 (or S100A9), could act as a potent endogenous ligand for TLR4 in various diseases including septic shock, vascular injury, and autoimmune disorders^12–14^. Besides, we reported that MRP8 expressed in macrophages (Mϕ) could be involved in the progression of diabetic nephropathy in mice and humans^6,15,16^. During these experiments, we unexpectedly observed that glomerular-infiltrated Mϕ expressed MRP8 much more robustly than tubulointerstitial Mϕ, which finding was also observed in human diabetic kidney and glomerulonephritis^16,17^. These observations suggested that the intraglomerular crosstalk may trigger and maintain high MRP8 expression in glomerular-infiltrated Mϕ. However, the mechanisms and roles for MRP8 upregulation in glomeruli remain elusive.

Because conventional systemic MRP8 deletion resulted in embryonic lethality^18^, MRP14 knockout mice were alternatively used in most of previous studies, where expression of MRP8 protein was at an undetectable level in myeloid cells^19^. MRP14 deletion in mice, however, does not recapitulate MRP8 deletion completely, as evidenced by no embryonic lethality in those mice. In the present study, we generated myeloid lineage cell-specific MRP8 knockout mice, and nephrotoxic serum-induced glomerulonephritis (NTN) was developed. We found that the ablation of MRP8 in myeloid cells exhibited less severe histological damage, proteinuria and inflammatory changes compared to control mice, and resulted in less M1 phenotype of Mϕ interacted with mesangial cells. Besides, the induction of macrophage-inducible C-type lectin (Mincle), which is critically involved in maintenance of M1 phenotype of macrophages^20^, was attenuated in monocytes-Mϕ and partly in granulocytes in MRP8-deleted NTN mice. The findings of this study suggested the role of MRP8 in myeloid cells might play important roles in the progression of crescentic glomerulonephritis by affecting Mϕ characterization.

## Results

### Effective recombination with reduction of MRP8 was achieved in myeloid cell-specific MRP8KO mice

We generated myeloid cell-specific MRP8-deficient (MyM8KO) mice, after confirming successful recombination (Supplemental Fig. S1B). We first evaluated the recombination efficiency in DNA, mRNA and protein levels (Supplemental Fig. S2). As previously reported^21^, lysozyme M-controlled Cre (LysM-Cre) recombinase mRNA was most abundantly expressed in bone-marrow cells, followed by leukocyte-rich organs such as the lung and spleen, but was almost absent in the liver, kidney or heart (Supplemental Fig. S2A). As for genomic DNA recombination, bone marrow and leukocyte-enriched organs, spleen and lung, showed a high efficiency as predicted, but the kidney and liver showed a low efficiency (Supplemental Fig. S2B,C). In the heart, unexpectedly, the targeted allele was also highly deleted by LysM-Cre recombinase. This result could be consistent with a previous report, which described that LysM-GFP was strongly and transiently expressed in embryonic cardiomyocytes^22^. In the mRNA level, LysM-Cre-mediated recombination led to 60–80% reduction of MRP8 in the bone marrow, lung and spleen. The kidney and liver also showed the reduced MRP8 level, which probably reflected a reduction of MRP8 in the infiltrated and/or residential myeloid-derived cells as in the lung and spleen (Supplemental Fig. S2D). Such reduction tendency was also confirmed in the protein level by Western blotting, which importantly indicated that myeloid lineage cell-specific MRP8 deletion resulted in markedly reduced serum MRP8 levels (Supplemental Fig. S2E).

### Upregulation and accumulation of MRP8 in the glomeruli of NTN were effectively attenuated in MyM8KO mice

**Figure 1:**
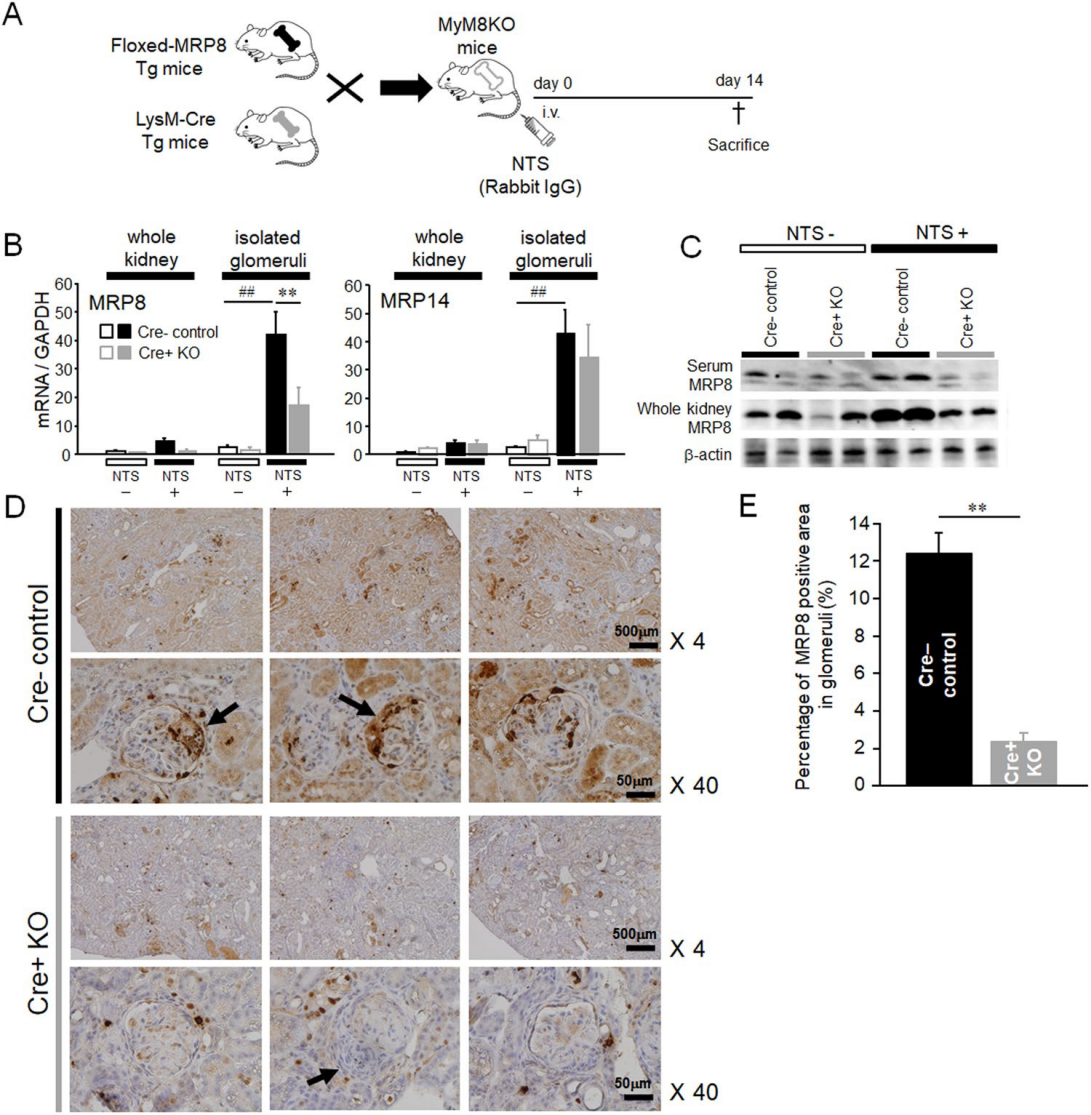
Induction of the experimental nephrotoxic glomerulonephritis (NTN) model in MyM8KO mice and MRP8 expression in this model. (**A**) To generate MyM8KO mice, floxed-MRP8 transgenic (Tg) mice was crossed with LysM-Cre Tg mice. (**B**) The mRNA expression of MRP8 and MRP14 was determined by TaqMan real-time RT PCR in the whole kidney and isolated glomeruli. Data are means ± SEM. n = 4–6, **P < 0.01 for Cre- control vs. Cre + KO. ^##^*P* < 0.01 for NTS- vs. NTS + . (**C**) The amount of MRP8 protein in the serum and kidney was evaluated by Western blotting. (**D**) Immunohistochemistry of MRP8 in Cre- control and Cre + KO NTN mice and (**E**) its quantification analysis of MRP8 positive area in glomeruli. Arrows indicate a crescentic lesion. Original magnification, x 4, x 40. Data are means ± SEM. n = 4–6, **P < 0.01 for Cre- control vs. Cre + KO. NTS, nephrotoxic serum; MRP8, myeloid-related protein 8; MRP14, myeloid-related protein 14.

To investigate the role of myeloid lineage cell-derived MRP8 in glomerulonephritis, we induced NTN in MyM8KO mice (Fig. 1A). After induction of NTN, MRP8 and its binding partner MRP14 were robustly upregulated especially in the isolated glomeruli rather than in whole kidney samples in Cre-negative control mice. Myeloid lineage cell-specific MRP8 deletion effectively suppressed MRP8 but not MRP14 (Fig. 1B). Such reduction was also observed in serum and the kidney tissue by Western blot analysis (Fig. 1C). In NTN mice, MRP8-positive signals were accumulated in glomerular-infiltrated Mac2-positive cells and crescentic lesions (Supplemental Fig. S3). MRP8 deletion significantly abolished these signals in the glomeruli (Fig. 1D,E). On the other hand, some tubular epithelial MRP8 signals remained elevated in MyM8KO mice (Fig. 1D). Faint staining of MRP8 that was broadly distributed in the tubules, possibly reflecting the reabsorption of MRP8 in the proximal tubules, was also decreased in MyM8KO mice. This finding might be compatible with reduced serum MRP8 concentrations in MyM8KO mice (Fig. 1C and Supplemental Fig. S2E).

### LysM-Cre-mediated recombination was effectively performed in MRP8-positive cells in NTN mice

**Figure 2:**
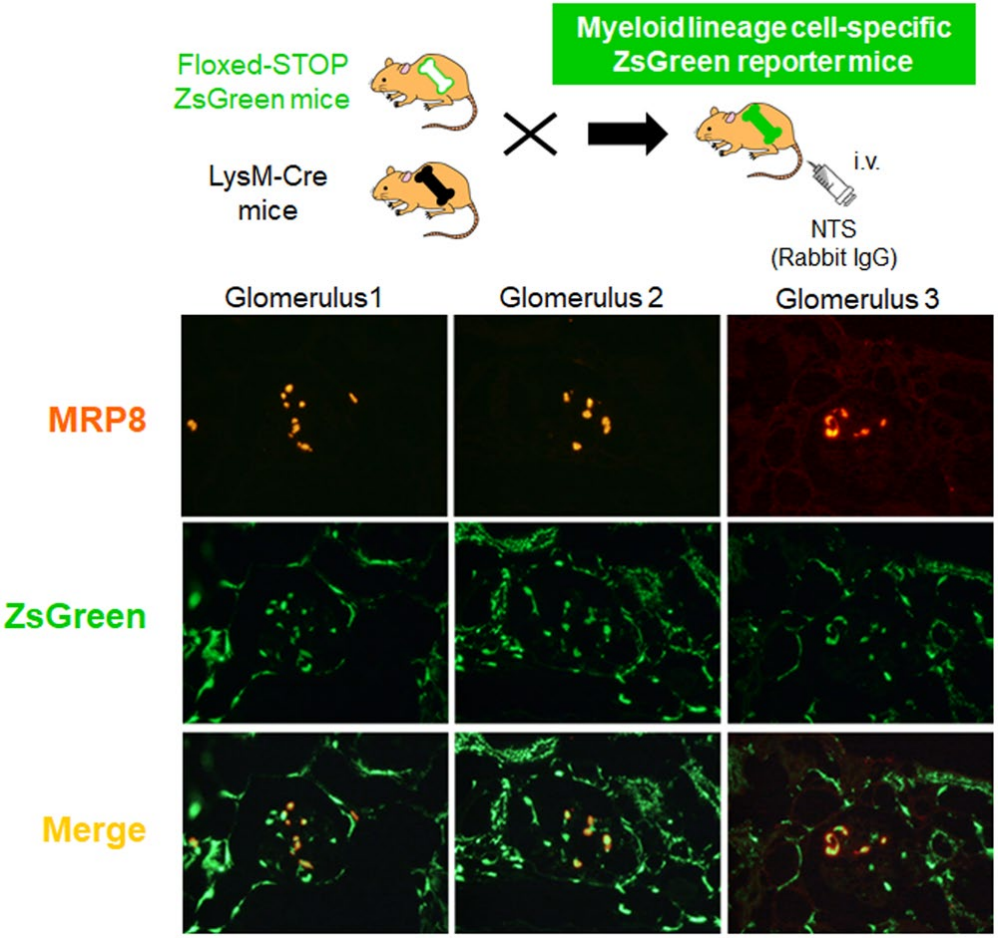
LysM-Cre-mediated recombination was visualized using myeloid lineage cell-specific ZsGreen reporter mice. Original magnification, x 40. LysM, Lysozyme M; NTS, nephrotoxic serum; MRP8, myeloid-related protein 8.

Next, we developed myeloid lineage cell-specific ZsGreen reporter mice by crossing floxed-STOP ZsGreen transgenic mice and LysM-Cre mice, and induced NTN in the reporter mice to examine the location of LysM-Cre-mediated recombination. As shown in Fig. 2, almost all glomerular MRP8 signals were included in ZsGreen signals, suggesting that LysM-Cre-mediated recombination was effectively performed in MRP8-positive cells in this model.

### Ablation of MRP8 in myeloid lineage cells ameliorated glomerulonephritis

**Figure 3:**
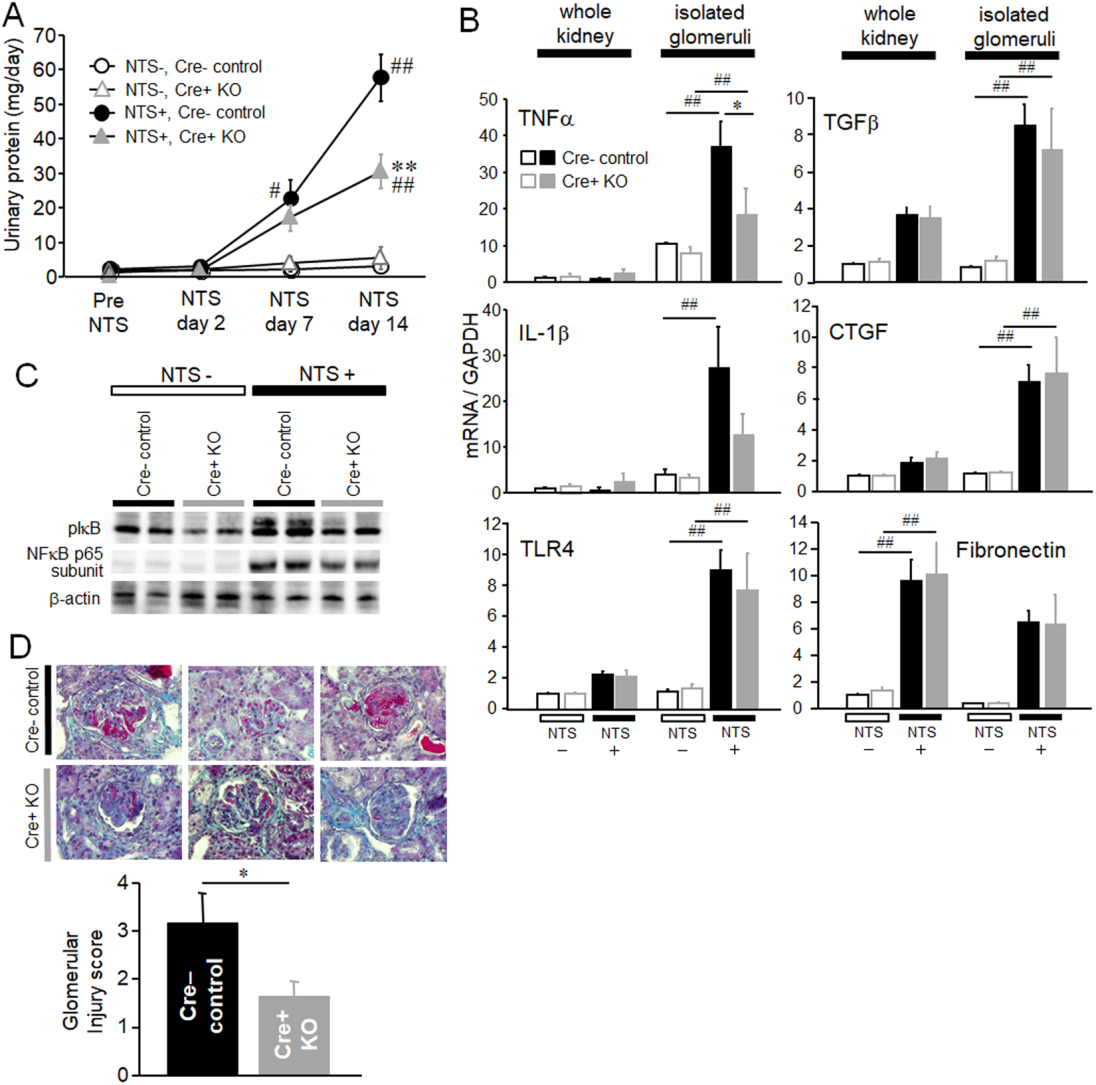
Effects of myeloid lineage cell-specific deletion of MRP8 on renal injuries in NTN mice. (**A**) Time course of urinary protein after induction of NTN. (**B**) The mRNA expressions of pro-inflammatory and pro-fibrotic genes and TLR4 determined by TaqMan real-time RT-PCR in the whole kidney and isolated glomeruli. (**C**) Western blotting of phospho-IκB and NFκB p65 subunit in the kidney. (**D**) Glomerular exudative lesion evaluated by Masson’s Trichrome staining and its quantified glomerular injury score. Original magnification, x 40. Data are means ± SEM. n = 4–6, **P < 0.01, **P < 0.01 for Cre- control vs. Cre + KO. ^#^*P* < 0.05, ^##^*P* < 0.01 for NTS- vs. NTS + . NTS, nephrotoxic serum; TNFα, tumor necrosis factor-alpha; IL-1β, interleukin-1beta; TLR4, toll-like receptor 4; TGFβ, transforming growth factor-beta; CTGF, connective tissue growth factor.

We next investigated the effects of myeloid lineage cell-specific MRP8 deletion upon renal injuries. MyM8KO mice revealed effective reduction in proteinuria, glomerular expression of TNFα and IL-1β, and the phosphorylation of IκB and NFκB p65 subunit after the induction of NTN (Fig. 3A–C). On the other hand, pro-fibrotic gene expressions, including TGFβ, CTGF and fibronectin, were almost comparable between Cre-negative control and MyM8KO mice (Fig. 3B). Deletion of MRP8 did not affect the expression of its receptor TLR4, which was upregulated markedly in the glomeruli of NTN mice (Fig. 3B). Importantly, glomerular exudative lesions evaluated by Masson’s trichrome staining were significantly diminished in MyM8KO mice (Fig. 3D).

### MRP8 expression in Mϕ was induced by co-culture with mesangial cells in a TLR4-independent manner

**Figure 4:**
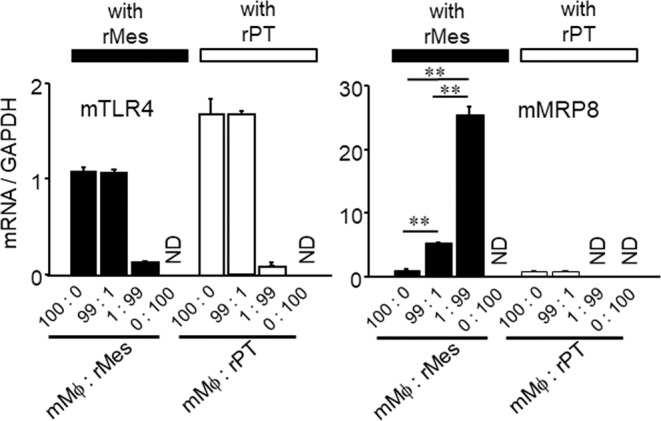
Effects of co-culture with renal intrinsic cells on TLR4 and MRP8 gene expressions in mouse Mϕ. Mouse TLR4 and MRP8 mRNA expression levels were determined by TaqMan real-time RT-PCR using mouse-specific probes. Data are means ± SEM. n = 4–8, **P < 0.01. mMϕ, RAW264.7 mouse macrophages; rMes, rat mesangial cells; rPT, rat proximal tubular cells; ND, not detected; mTLR4, mouse toll-like receptor 4; mMRP8, mouse myeloid-related protein 8.

Because the MRP8 positivity in Μϕ was apparently higher in glomerular-infiltrated Μϕ compared to tubulointerstitial Μϕ in NTN mice (Figs. 1B,D and 2), we performed co-culture experiments to investigate the potential intraglomerular cell-cell crosstalk affecting MRP8 and TLR4 expressions in Mϕ. The TLR4 mRNA expression in mouse Mϕ (mMϕ) was not affected by co-culture with either rat mesangial cells (rMes) or proximal tubular cells (rPT), and its expression levels appeared to just reflect the proportion of mMϕ (Fig. 4, left). Similarly, the MRP8 mRNA expression in mMϕ was not changed by co-culture with rPT; when co-cultured with rMes, however, the MRP8 gene expression in mMϕ was induced (Fig. 4, right). Such induction was recapitulated by stimulation with rMes-cultured supernatant (Supplemental Fig. S4). Because we and others reported that MRP8 could be induced in Mϕ in a TLR4-dependent manner^12,16^, we pharmacologically examined whether the induction of MRP8, and possibly of other pro-inflammatory cytokines, by mesangial cells *in vitro* was also TLR4-dependent by using E5564, a TLR4 antagonist. The upregulation of MRP8, IL-1β and TNFα by the rMes-cultured medium was not or minimally affected by the treatment with E5564 (Supplemental Fig. S5), suggesting that the induction of MRP8 and other cytokines induced by humoral factors from mesangial cells could be TLR4-independent. Similarly, there was no effect of E5564 on cytokine expression in rPT-cultured medium-treated Mϕ (Supplemental Fig. S6).

### Myeloid lineage cell-specific deletion of MRP8 revealed less M1 dominancy in Mϕ

**Figure 5:**
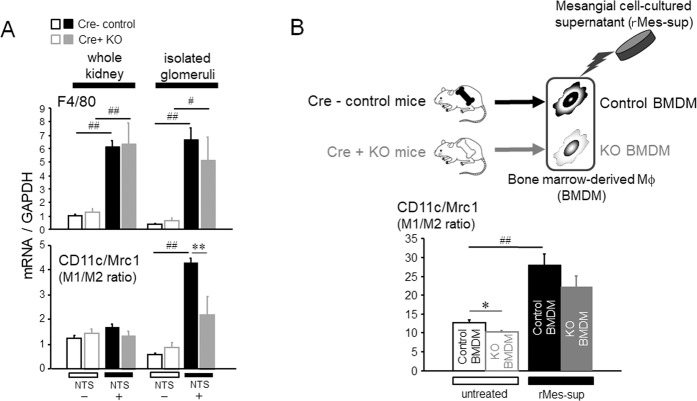
Effects of myeloid lineage cell-specific MRP8 deletion on Mϕ polarization *in vivo* and *in vitro*. (**A**) In the kidney of NTN mice, F4/80 mRNA levels and the M1/M2 ratio defined by the CD11c/Mrc1 ratio were evaluated. n = 4–6, **P < 0.01 for Cre- control vs. Cre + KO. ^#^*P* < 0.05, ^##^*P* < 0.01 for NTS- vs. NTS + . (**B**) BMDM, which were generated from Cre-negative control and from Cre-positive KO mice, were stimulated with rMes-sup for 24 hours. The M1/M2 ratio was evaluated. n = 4, *P < 0.05 for Control BMDM vs. KO BMDM. ^##^*P* < 0.01 for untreated vs. rMes-sup-treated. Data are means ± SEM. NTS, nephrotoxic serum; BMDM, bone-marrow derived macrophages; Mrc1, mannose receptor C type 1.

After induction of NTN, a pan-Mϕ marker F4/80 was markedly increased in isolated glomeruli but was minimally affected by MRP8 deletion in Mϕ, whereas the M1/M2 ratio defined by CD11c/Mrc1 was significantly suppressed in MyM8KO mice (Fig. 5A). Then, in order to examine the effects of MRP8 deletion upon Mϕ characterization, we generated bone marrow-derived Mϕ (BMDM) from Cre-negative control (Control BMDM) and from MyM8KO mice (KO BMDM), and stimulated them with rMes-cultured medium. The treatment resulted in the increased M1/M2 ratio in Control BMDM; however, KO BMDM showed less M1 dominancy upon stimulation (Fig. 5B). Importantly, more robust stimulation by LPS with skewing toward M1 dominancy in Control BMDM showed an apparently less response in KO BMDM (Supplemental Fig. S7). In addition, we examined mRNA expression levels of interleukin-10 (IL-10) as another Mϕ M2 marker, CD4/CD8 ratio as a surrogate marker of T lymphocyte balance, and the receptor for advanced glycation end products (RAGE) as another pattern recognition receptor for MRP8. MyM8KO mice showed a significant suppression of IL-10 mRNA upregulation in the glomeruli after induction of NTN, while there was no difference in the CD4/CD8 ratio or RAGE expression between the genotypes (Supplemental Fig. S8).

### Deletion of MRP8 resulted in less stress fiber formation in Mϕ

**Figure 6:**
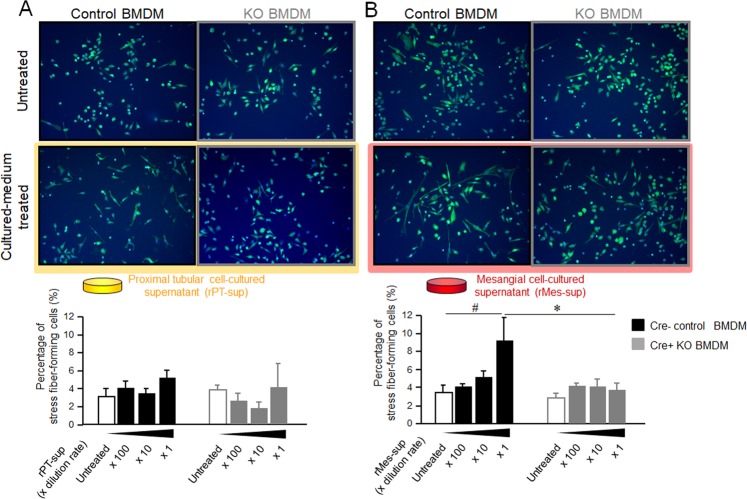
Impact of MRP8 deletion on the stress fiber formation induced by Mes-sup stimulation in BMDM. BMDM, which were generated from Cre-negative control and from Cre-positive KO mice, were incubated with rPT-sup (**A**) or rMes-sup (**B**) for 24 hours. Percentage of stress fiber-forming cells was quantified. Data are means ± SEM. n = 4–5, *P < 0.05 for Control BMDM vs. KO BMDM. ^#^*P* < 0.05 for untreated vs. rMes-sup-treated.

Next, we evaluated stress fiber formation in Mϕ by phalloidin staining. Stress fiber-forming cells were defined as cells stretched over 100 μm (Supplemental Fig. S9). The rPT-cultured medium did not affect the stress fiber formation both in Control BMDM and KO BMDM (Fig. 6A). On the other hand, rMes-cultured medium significantly increased the occurrence of stress fiber-forming cells in Control BMDM, and this effect was effectively abrogated by MRP8 deletion in KO BMDM (Fig. 6B).

### Mincle expression on monocytes- Mϕ was attenuated by MRP8 ablation in NTN mice

**Figure 7:**
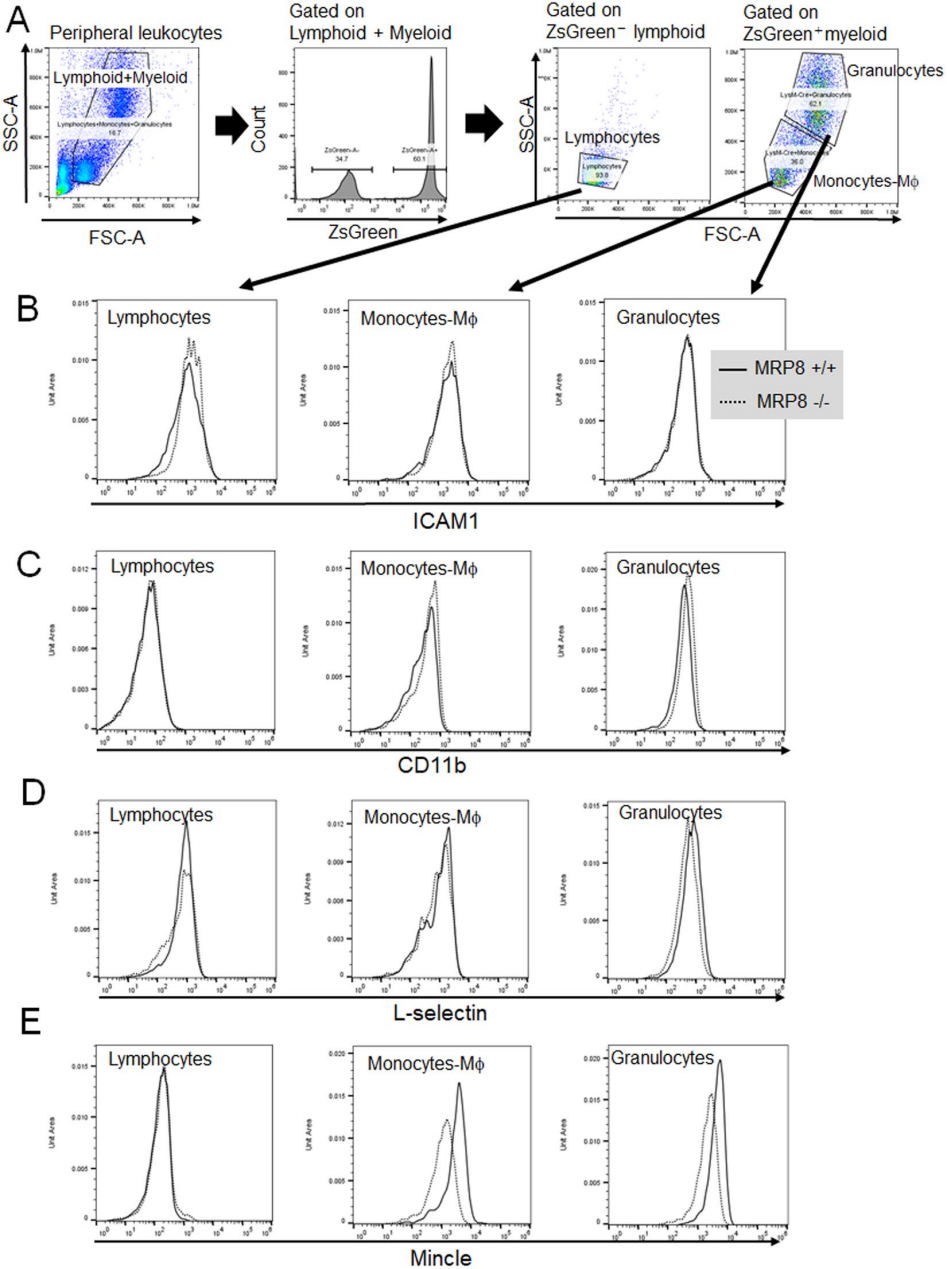
Flow cytometry (FCM) of peripheral blood cells in MyM8KO NTN mice. (**A**) Sorting strategy for lymphocytes, monocytes and granulocytes in blood. Peripheral leukocytes were separated into lymphoid and myeloid by ZsGreen. Monocytes-Mϕ and granulocytes were gated by the conventional method in FSC-SSC plot. (**B**-**E**) FCM of ICAM1, CD11b, L-selectin and Mincle in peripheral leukocytes. Solid and dotted lines show the results in flox-negative control and myeloid lineage cell-specific MRP8 deleted mice, respectively.

To examine whether MRP8 deletion in myeloid lineage cells affects the Mϕ cell surface markers involved in adhesion and/or migration, FCM analyses were performed for leukocytes in MyM8KO mice (Fig. 7A and Supplemental Fig. S10A). In non-NTN healthy mice, there were no apparent differences in the expression of the markers examined between the genotypes (Supplemental Fig. S10B-E). It is noteworthy that the induction of Mincle expression was effectively attenuated in monocytes-Mϕ, and partly in granulocytes, from MyM8KO NTN mice (Fig. 7E). In contrast to Mincle, no difference was observed in the expression of L-selectin, another C-type lectin (Fig. 7D).

## Discussion

In the present study, we demonstrated the crucial role of MRP8 expressed in myeloid cells for the development and progression of glomerulonephritis in mice. So far, accumulating evidence has revealed the importance of endogenous molecules released from the cells upon damages or dangers, i.e., DAMPs, in the pathophysiology of a variety of diseases^3–5^. Among them, the role of a heterodimer MRP8/MRP14 has been shown in multiple situations including autoimmune disease^14^, cancer^23^, atherosclerotic disease^13,24,25^, and kidney injury^26^. However, all of these studies were performed using the mice deficient of MRP14, a binding partner of MRP8. To our knowledge, the present study will provide the first direct evidence that loss of MRP8 in myeloid cells could significantly lessen the organ damage *in vivo*. Furthermore, our data revealed that MRP8 deletion affected the characterization of glomerular-infiltrated cells and peripheral blood Mϕ upon glomerulonephritis. Importantly, enhanced Mincle expression was significantly alleviated in Mϕ and partly in neutrophils by the absence of MRP8.

In our experiments, Mϕ M1 phenotype was attenuated in parallel with the suppression of gene expression of pro-inflammatory cytokines and the NFκB signaling. In contrast, the expression of Mrc1 and Arginase1 (data not shown), the M2 markers, was maintained, similar to that of pro-fibrotic genes such as TGFβ, CTGF or fibronectin (Fig. 3). In this regard, it is important to note that M2 Mϕ or fibrocytes could be involved in the development and maintenance of kidney fibrosis^26–28^. Nevertheless, it is also important to note that the early and proper intervention of inflammation with immunosuppressants during an acute phase is critical for the treatment of crescentic glomerulonephritis such as anti-GBM or ANCA-associated glomerulonephritis. In the present study, MyM8KO mice showed the suppression of M1 markers and pro-inflammatory cytokines, along with unaltered, but not aggravated, M2 markers and pro-fibrotic genes after induction of NTN. On the other hand, MyM8KO mice showed significant suppression of IL-10 mRNA expression, which induces the M2 Mϕ differentiation, in the glomeruli of NTN (Supplemental Fig. S8). To note, however, there is a heterogeneity of Mϕ population involved in kidney injury, being not simply divided into M1 and M2 phenotype. So, we have to consider the diversity including an alternatively activated subset of M2 Mϕ rather than just M2 dominancy^29,30^, and certain changes of Mϕ characterization by MRP8 deletion could possibly suppress pro-inflammatory reaction without influence on pro-fibrotic changes in the acute phase of NTN.

We and others reported that MRP8 was upregulated in leukocytes in the diabetic milieu^6,31^, the upregulation of which could be induced by glucolipotoxicity in a TLR4-dependent manner and may represent a positive autocrine loop^6,15^. In the present study, however, the induction of MRP8 in Mϕ by mesangial cells appeared to occur in a TLR4-independent manner. MRP8 could also reportedly interact with RAGE^23,32^. Therefore, we examined RAGE expression in the kidney of NTN mice. The expression of RAGE increased dominantly in the isolated glomeruli, just similar to that of TLR4 (Supplemental Fig. S8). The involvement of RAGE in this amelioration could not be excluded in the glomerular lesion at present. Nevertheless, our findings suggest a possibility that the inhibition of MRP8, rather than blockade of its receptor TLR4, could provide another strategy along with additional effects via the RAGE pathway. Another important issue is its local induction upon kidney disease. In our study, MRP8 was robustly and locally induced in the injured glomeruli. Actually, it is reported that glomerular MRP8-positive cells were accumulated in various glomerular diseases such as ANCA-associated glomerulonephritis^16^, IgA nephritis^33^ or membrano-proliferative glomerulonephritis^34^. Vogl T, *et al*. reported that MRP8/MRP14 could indicate the local inflammation in very early phase of the diseases and the prognosis of late disease activities in a model of contact dermatitis or collagen-induced arthritis^35^. Hence, the evaluation of local induction of MRP8 in the kidney tissue would become a useful tool in monitoring renal local inflammation.

There is a variety of cells participating in and contributing to the pathogenesis and progression of crescentic glomerulonephritis. Mϕ are typically involved in the subacute to chronic process following the initial endothelial injury with neutrophil and T cell activation, leading to glomerular exudative lesion^36,37^. In the present study, we found no evidence that loss of MRP8 in myeloid lineage cells affects the lymphocyte characteristics detected by FCM and real-time PCR (Fig. 7, Supplemental Figs. S8 and S10). On the other hand, some of surface molecules affecting adhesion and/or migration were altered in Mϕ and also in neutrophils. Especially, we revealed that Mincle could be an important molecule whose upregulation upon NTN was attenuated by MRP8 deletion. Mincle is a member of the C-type lectin receptor family bound to carbohydrates in a calcium-dependent manner. It has been shown that Mincle plays a pivotal role as a pattern recognition receptor, amplifying innate immunity and activating Mϕ and neutrophils by sensing adjuvant glycolipids, some pathogens and DAMPs released from dying cells^38,39^. It is also recently reported that Mincle is involved in the maintaining M1 phenotype in kidney injury^20^. Although MRP8 induces neutrophil chemotaxis and adhesion by activating L-selectin, another C-type lectin^40^, we could not find any effect on L-selectin by MRP8 deletion. The present study suggested the role of MRP8 in affecting Mincle expression on Mϕ and also weakly on neutrophils. Meanwhile, focusing on the glomerular intrinsic cells, we demonstrated a novel role of mesangial cells in glomerular injury through enhancing MRP8 expression and the following characterization changes in Mϕ. We previously reported that mesangial cells could be important in Mϕ infiltration during glomerulonephritis in a CTGF-dependent manner^41^. Therefore, we focused on the intraglomerular crosstalk between mesangial cells and Mϕ, which induces the expression of MRP8 and M1 dominancy which results in aggravation of kidney injury. Of course, the other glomerular intrinsic cells, especially glomerular endothelial cells, should be noted to interact with Mϕ in progression of glomerulonephritis^42^.

It should be considered carefully that a lysozyme M promoter is not restricted to Mϕ but can work in other myeloid lineage cells^43^. MRP14 deletion, resulting in loss of circulating MRP8, is shown to differentially modify phenotypic states of neutrophils, Mϕ, and dendritic cells in atherosclerosis or adipose tissue inflammation^44^. Hence, we could not deny the possibility that MRP8 in neutrophils contributes to the pathophysiology in NTN. In fact, MRP8-targeting strategies are proposed in various pathological conditions related to the neutrophil activation^25,32,44,45^.

In summary, the present study revealed that MRP8 deletion in myeloid lineage cells resulted in ameliorated glomerular lesions and less M1 phenotype with reduced Mincle expression in a mouse model of crescentic glomerulonephritis. Our data indicate that MRP8 in myeloid cells could potentially aggravate glomerular injury through intraglomerular cell-cell crosstalk affecting Mϕ characterization, suggesting the suppression of MRP8 as a potential therapeutic target to ameliorate crescentic glomerulonephritis.

## Methods

### Experimental animals

To generate tissue-specific MRP8 knockout mice, we employed the conventional Cre-loxP system. To flank exons 2 and 3 of the mouse S100a8 gene, which encode a whole coding sequence of MRP8 by loxP sites (MRP8^*fl/fl*^), we used homologous recombination in embryonic stem cells (for more details, see Supplemental information Supplemental Fig. S1). We developed myeloid lineage cell-specific MRP8 knockout mice (MyM8KO) by crossing MRP8^*fl/fl*^ and lysozyme M-Cre (LysM-Cre) mice, which were purchased from the Jackson Laboratory (Bar Harbor, ME, USA). B6.Cg-Gt(ROSA)26Sor < tm6(CAG-ZsGreen1)Hze >/J mice (Jackson Laboratory) were also crossed with LysM-Cre mice to develop myeloid lineage cell-specific ZsGreen reporter mice. Recombination efficacy of MyM8KO mice was evaluated by DNA, mRNA and protein levels with PCR, RT-PCR and Western blotting, respectively. Sequences of PCR primers were 5′-TTCTAGCAGTGTCTAGCAGAAGAGG-3′ (forward) and 5′-GAGACCATGTATTTGAGAGGCAGTT-3′ (reverse). All animal experiments were approved by Animal Research Committee of Kumamoto University (Certification No. G26-120) along with guidelines. Besides, all experiments were performed in accordance with relevant guidelines and regulations.

### Induction of the experimental nephrotoxic glomerulonephritis

Experimental NTN was induced by the conventional method as we previously reported^46^. Briefly, glomeruli were isolated by differential sieving from the ddY mouse renal cortex and disrupted by sonication. The glomerular basement membrane (GBM) was collected by centrifugation, emulsified with complete Freund’s adjuvant (CFA; Difco, Detroit, MI, USA) and immunized in rabbits. Mice were immunized by an intraperitoneal injection of 1.0 mg per 20 g body weight of normal rabbit IgG (ICN, Aurora, OH, USA) emulsified with CFA. Five days later, 0.3 ml per animal of anti-GBM antiserum (nephrotoxic serum [NTS]) or isovolume of control normal rabbit serum was injected from the tail vein. Urine samples were collected with metabolic cages for 24 hours, and urinary protein was measured by the pyrogallol red-molybdate protein dye-binding method (SRL, Tokyo, Japan). Mice were sacrificed, and all tissues were collected at day 14 after induction of glomerulonephritis.

### Real-time quantitative RT-PCR

Total RNA was extracted with TRIzol reagent (Invitrogen, Carlsbad, CA, USA) and cDNA in each sample was synthesized by High Capacity cDNA Reverse Transcription Kit (Applied Biosystems, Foster City, CA, USA) from mouse kidneys and glomeruli that were isolated by graded sieving method^6,46^. TaqMan real-time PCR was performed using Premix Ex Taq (Takara Bio, Otsu, Japan) and StepOnePlus Real Time PCR System (Applied Biosystems) (see Supplemental information Supplemental Table S1 for primer and probe sequences). Expression levels of all genes were normalized by *Gapdh* (internal control) levels which were detected with TaqMan Rodent GAPDH Control Reagents (Applied Biosystems). The mean expression level in the whole kidney of Cre-negative, non-NTS-treated control mice was arbitrarily defined as 1.0.

### Histological analysis

Masson’s Trichrome staining and immunohistochemistry of MRP8 (requiring antigen retrieval by citrate buffer) and Mac-2 (or Lgals3)^6^ were carried out using kidney sections (thickness 4 μm) fixed with 4% buffered paraformaldehyde. Nuclei were counterstained with hematoxylin. All primary antibodies used in this study are shown in Supplemental information Supplemental Table S2. For double staining, the primary antibody for MRP8 was visualized with a DyLight-conjugated secondary antibody (Jackson ImmunoResearch, PA, USA). Immunofluorescence of MRP8 evaluating colocalization with ZsGreen signals was performed with snap frozen cryostat sections (4 μm), prefixed with 4% buffered paraformaldehyde, and with the primary antibody. Photos were taken by a fluorescence microscope (IX81-PAFM; Olympus, Tokyo, Japan). Glomerular exudative lesion, which is defined by the red-colored lesion in Masson’s Trichrome staining with extracapillary inflammatory-cells accumulation, of more than 10 glomeruli were measured quantitatively to obtain an average for each mouse using MetaMorph 7.5 software (Molecular Devices, Downingtown, PA, USA). Actually, there were various degrees of exudative lesion in each glomerulus. Therefore, a glomerular injury score was used to assess the degree of glomerular damage as follows: score 0, 0% glomerular exudative area (% of glomerulus); 1, 0–1%; 2: 1–3%; 3, 3–6%; 4, 6–10%; 5, 10–20%; 6, over 20%.

### Western blot analysis

Proteins extracted from kidney samples were separated by SDS-PAGE, transferred onto PVDF membranes, incubated with primary antibodies and detected with peroxidase-conjugated secondary antibodies and chemiluminescence^6,47^. Gapdh or β-actin was used as an internal control. All primary antibodies used in this study are shown in Supplemental information Supplemental Table S2.

### Flow cytometry (FCM) for leukocytes

For effective sorting of MRP8-targeted cells, MyM8KO mice were crossed with B6.Cg-Gt(ROSA)26Sor < tm6(CAG-ZsGreen1)Hze >/J mice (MyM8KO-ZsG). MyM8KO-ZsG mice enabled us to detect LysM-Cre-induced MRP8-deleted cells with ZsGreen1.

Peripheral blood was collected, and red blood cells were lysed by FCM lysing solution (Santa Cruz Biotechnology, Santa Cruz, CA, USA). White blood cells were collected by centrifugation at 300 x g for 5 minutes and resuspended in cold PBS. The cells were incubated with anti-CD16/CD32 for 10 minutes in the dark to block nonspecific binding of antibodies to the FcγIII/II receptors followed by staining with surface markers. Cells were analyzed on the SH800S Cell Sorter (Sony Biotechnology, Tokyo, Japan). Dead cells were excluded by 4′,6-diamidino-2-phenylindole (DAPI) labelling. All primary antibodies used in this study are shown in Supplemental information Supplemental Table S2.

### Cell culture

Murine Mϕ cell line RAW264.7 (mMϕ) and rat renal proximal tubular cell line NRK52E (rPT) were purchased from ATCC (Manassas, VA, USA). Rat mesangial cells (rMes) were kindly provided by Dr. Daisuke Nakano and Prof. Akira Nishiyama (Department of Pharmacology, Kagawa University Medical School, Kagawa, Japan). They were isolated from male Sprague-Dawley rats and were maintained according to previous publications^48,49^. RAW264.7 Mϕ were co-cultured with rPT or rMes. Total mRNA was extracted after co-culture for 72 hours, and mRNA expression levels were determined by TaqMan real-time PCR. To detect the gene expression levels of Mϕ, TaqMan probes were designed to be mouse-specific complementary sequences. We generated bone marrow-derived Mϕ (BMDM) from Cre-negative control and Cre-positive MyM8KO mice to establish the MRP8-deficient Mϕ. BMDM were generated from mice as described previously^50^. Briefly, following lysis of red blood cells, bone marrow cells were resuspended in medium containing 20% fetal calf serum and 50 ng/ml recombinant murine GM-CSF (Peprotech, Rocky Hill, NJ, USA), and cultured at 37 °C in 5% CO_2_ atmosphere. On day 6, medium was replaced with serum-free medium. On day 7, BMDM were used for each experiment. To evaluate the effects of MRP8 deletion in Mϕ, BMDM were stimulated with rMes-cultured medium or lipopolysaccharide (LPS; Sigma-Aldrich, St Louis, MO, USA). Stress fiber formation in Mϕ was evaluated by phalloidin staining. BMDM were fixed with 4% paraformaldehyde, permeabilized and immunostained with FITC-phalloidin (Sigma Aldrich, St Louis, MO, USA) to visualize actin filaments. Stress fiber-forming cells were defined as cells stretched over 100 μm. E5564 (Eisai Inc., Woodcliff Lake, NJ, USA) is a synthetic analog of lipid A and a potent and specific antagonist of TLR4, which terminates MD2/TLR4-mediated signaling^51^. To test the effects of E5564 on Mϕ, RAW264.7 Mϕ were stimulated with rMes-cultured supernatant (rMes-sup) or rPT-cultured medium (rPT-sup) for 24 hours. E5564 was added simultaneously with rMes-sup or rPT-sup stimulation. Total RNA was extracted with RNeasy mini kit (Qiagen, Tokyo, Japan).

### Statistical analysis

Data are expressed as means ± SEM. Differences between multiple groups were assessed by two-way factorial ANOVA with Bonferroni’s post-hoc test. Comparison between two groups was carried out by unpaired Student’s t test. Statistical significance was defined as *p* < 0.05.

## Supplementary information

Supplementary information.
