# Supplementary material for: Ablation of Myeloid Cell MRP8 Ameliorates Nephrotoxic Serum-induced Glomerulonephritis by Affecting Macrophage Characterization through Intraglomerular Crosstalk
Source: Sci Rep. 2020 Feb 20;10:3056. doi: 10.1038/s41598-020-59970-9 (PMC7033179; doi:10.1038/s41598-020-59970-9)

Online Supplementary Information

Title

Ablation of Myeloid Cell MRP8 Ameliorates Nephrotoxic Serum-induced Glomerulonephritis by Affecting Macrophage Characterization through Intraglomerular Crosstalk

Yusuke Hata*,[1] Takashige Kuwabara*,[1†,2] Kiyoshi Mori,[2,3†,4†] Youngna Kan,[2] Yuki Sato,[2] Shuro Umemoto,[1] Daisuke Fujimoto,[1] Tomoko Kanki,[1] Yoshihiko Nishiguchi,[1] Hideki Yokoi,[2] Yutaka Kakizoe,[1] Yuichiro Izumi,[1] Motoko Yanagita,[2,5] and Masashi Mukoyama[1†,2]

[1]Department of Nephrology, Kumamoto University Graduate School of Medical Sciences, Kumamoto, Japan.

[2]Department of Nephrology, Kyoto University Graduate School of Medicine, Kyoto, Japan.

[3]Department of Nephrology and Kidney Research, Shizuoka General Hospital, Shizuoka, Japan.

[4]School of Pharmaceutical Sciences, University of Shizuoka, Shizuoka, Japan.

[5]Institute for the Advanced Study of Human Biology (ASHBi), Kyoto University, Kyoto, Japan

*Equally contributed

[†]Present Address

Full Methods

Generation of Myeloid lineage cell-specific MRP8 knockout mice

MRP8 genomic DNA was isolated from BAC clone RP23-190D1 or RP23-6E18 containing C57BL/6 genomic sequence including the MRP8 gene. From this BAC a 9.5-kb genomic fragment digested by *Sac*I was subcloned and used to generate a conditional MRP8 targeting vector. The targeting vector was designed so that exons 2 and 3, which contain the start codon, were inserted behind the 5' loxP site followed by FRT-flanked neomycin resistance cassette (Supplemental Figure S1A). An ES clone of C57BL/6 carrying the targeted allele was obtained under G418 selection (Supplemental Figure S1B). Finally, the floxed-MRP8-transgenic mice were established by crossing with FLP-transgenic mice to delete neomycin resistance cassette.

Supplemental Figure Legends

Supplemental Figure S1

(A) Generation of a conditional MRP8 gene. Conditional gene targeting strategy for the disruption of the mouse MRP8. (B) PCR confirmation of targeted ES clone establishment. An ES clone carrying the targeted allele was obtained under G418 selection (lane 4). Cre-expressing plasmid was transfected to the targeted ES clone to delete Exon and Neo regions, indicating that Exon removal was possible in vitro (lane 2).

Supplemental Figure S2

(A) Evaluation of recombination efficiency in MyM8KO mice. mRNA expression of LysM promoter-driven Cre recombinase was determined by TaqMan real-time RT-PCR in LysM-Cre transgenic mice. (B, C) LysM-Cre-mediated genomic DNA recombination was examined by PCR using primer pairs which were specifically designed to detect the wild type, undeleted and deleted alleles. (D, E) The expression levels of MRP8 mRNA and protein were evaluated by TaqMan real-time RT-PCR and Western blot analysis, respectively. LysM, lysozyme M; KO, knockout.

Supplemental Figure S3

Double immunostaining of Mac2 and MRP8 in NTN mice. White arrows indicate the MRP8-positive cells and their locations.

Supplemental Figure S4

Effects of stimulation with cultured medium of renal intrinsic cells on TLR4 and MRP8 gene expressions in RAW264.7 mouse Mϕ. Mϕ were incubated with cultured medium of proximal tubular cells or mesangial cells for 24 hours, then total mRNA was collected. Data are means ± SEM. n=4, ** $P<0.01$. Mϕ, macrophages; rMes-sup, supernatant of rat mesangial cells; rPT-sup, supernatant of rat proximal tubular cells; mTLR4, mouse toll-like receptor 4; mMRP8, mouse myeloid-related protein 8.

Supplemental Figure S5

Effects of E5564, a TLR4 antagonist, on Mϕ treated with mesangial cell-cultured medium. RAW264.7 (mMϕ) cells stimulated with rMes-sup were incubated with E5564 for 24 hours. Data are means ± SEM. n=4-5, *P<0.05, **P<0.01. rMes-sup, rat mesangial cell-cultured supernatant; IL-1β, interleukin-1 beta; TNFα, tumor necrosis factor-alpha; MRP8, myeloid-related protein 8; TLR4, toll-like receptor 4.

Supplemental Figure S6

Effects of E5564, a TLR4 antagonist, on Mϕ treated with proximal tubular cell-cultured medium. RAW264.7 mouse Mϕ stimulated with rPT-sup were incubated with E5564 for 24 hours. Data are means ± SEM. n=4, *P<0.05. Mϕ, macrophages; rPT-sup, rat proximal tubular cell-cultured supernatant; IL-1β, interleukin-1 beta; TNFα, tumor necrosis factor-alpha; MRP8, myeloid-related protein 8; TLR4, toll-like receptor 4.

Supplemental Figure S7

Effects of MRP8 deletion on LPS-induced pro-inflammatory phenotypic changes in Mϕ. BMDM were stimulated with LPS for 40 hours. Data are means ± SEM. n=3-4, $^{\#}P$<0.05, $^{\#\#}P$<0.01 among different LPS concentrations in Control BMDM, $^{\$\$}P$<0.01 among different LPS concentrations in KO BMDM, **P<0.01 for Control BMDM vs. KO BMDM. LPS, lipopolysaccharide; Mϕ, macrophages; BMDM, bone marrow-derived macrophages; Mrc1, mannose receptor C type 1.

Supplemental Figure S8

Effects of myeloid lineage cell-specific deletion of MRP8 on mRNA expressions by TaqMan real-time RT-PCR in the whole kidney and isolated glomeruli. Data are means ± SEM. n=4-6, **P<0.01, **P<0.01 for Cre- control vs. Cre+ KO. $^{\#\#}P$<0.01 for NTS- vs. NTS+. NTS, nephrotoxic serum; IL-10, interleukin-10; RAGE, receptor for advanced glycation end product.

Supplemental Figure S9

Evaluation of stress fiber formation in BMDM by phalloidin staining. (A) BMDM, which were generated from Cre-negative control and from Cre-positive KO mice, were incubated with rMes-sup or rPT-sup for 24 hours. (B) Stress fiber-forming cells were defined as cells stretched over 100 μm as indicated with white triangles. BMDM, bone marrow-derived macrophages.

Supplemental Figure S10

Flow cytometry (FCM) of leukocytes in myeloid lineage cell-specific MRP8 deleted non-NTN, healthy mice. (A) Sorting strategy for lymphocytes, monocytes and granulocytes in blood. Peripheral leukocytes were separated into lymphoid and myeloid by ZsGreen. Monocytes-Mϕ and granulocytes were gated by the conventional method in FSC-SSC plot.

(B-E) FCM of ICAM1, CD11b, L-selectin and Mincle in peripheral leukocytes. Solid and dotted lines show the results in flox-negative control and myeloid lineage cell-specific MRP8 deleted mice, respectively.

Supplemental Tables

Supplemental Table S1. Sequences of primers and probes used in this study

Gene	Forward primer	Reverse primer	Probe
F4/80	5'-TGGTGGTCATAATCTCTGCTTCTG-3'	5'-AGACAGGCCCCAGGAAACTC-3'	5'-FAM-CCCGTCTCTGTATTCAACCAGCAGCGATT-TAMRA-3'
MCP-1	5'-TTGGCTCAGCCAGATGCA-3'	5'-CCAGCCTACTCATTGGGATCA-3'	5'-FAM-CCCCACTCACCTGCTACTCATTCA-TAMRA-3'
TNFa	5'-AAGGCTGCCCCGACTACG-3'	5'-AGGTTGACTTTCTCCTGGTATGAG-3'	5'-FAM-AGGTTGACTTTCTCCTGGTATGAG-TAMRA-3'
IL-1b	5'-TCGTGCTGTCGGACCCATA-3'	5'-ACAGGTATTTTGTCGTTGCTTGG-3'	5'-FAM-AGCTGAAAGCTCTCCACCTCAATGGACA-TAMRA-3'
TGFb	5'-GACGTCACTGGAGTTGTACGG-3'	5'-GCTGAATCGAAAGCCCTGT-3'	5'-FAM-AGTGGCTGAACCAAGGAGACGGAA-TAMRA-3'
Fibronectin	5'-ATCATTTCATGCCAAGTT-3'	5'-TCGCACTGGTAGAAGTTCCA-3'	5'-FAM-CCGACGAAGAGCCCTTACAGTTCCA-TAMRA-3'
CTGF	5'-TTCCCGAGAAGGGTCAAGCT-3'	5'-TCCTTGGGCTCGTCACACA-3'	5'-FAM-CCTGGGAAATGCTGCAAGGAGTGG-TAMRA-3'
TLR4	5'-CTTCAGTGGCTGGATTTATC-3'	5'-GAGGTGGTGTAAGCCATG-3'	5'-FAM-CATGCCTTGTCTTCAATTGTTTCAA-TAMRA-3'
RAGE	5'-CTGCTCGTCGGGGCTATCC-3'	5'-CTCTGACTGATTCAGCTCTGCAC-3'	5'-FAM-CCTTCCTCTCCTCACGCCTGGGTTGT-TAMRA-3'
MRP8	5'-ATCCTTTGTCAGCTCCGTCTTC-3'	5'-GGGCATGGTGATTTCCTTGTATATT-3'	5'-FAM-ATCTTTCGTGACAATGCCGTCTGAACTGGA-TAMRA-3'
MRP14	5'-GAAGGAAGGACACCCTGACAC-3'	5'-GAGAAGAGAAATGAAGCCCTCATAAA-3'	5'-FAM-AATGGTGGAAGCACAGTTGGCAACCTT-TAMRA-3'
HMGB1	5'-GGACCCCAATGCACCCAAG-3'	5'-TCACCAATGGATAAGCCAGGATG-3'	5'-FAM-CCTCCTTCGGCCTTCTTCTTGTTCTGTTCT-TAMRA-3'
CD4	5'-CTGACTCTGACTCTGGACAAAGG-3'	5'-GGAGAGGTAGGTCCCATCACC-3'	5'-FAM-TTGAGCTGAGCCACTTTCATCACCACCA-TAMRA-3'
CD8a	5'-GCCCCAGAGACCAGAAGATTG-3'	5'-GTGCCCAGATGTAAATATCACAGG-3'	5'-FAM-CGGTCCCCTTCACTGAGCCACGG-TAMRA-3'
CD11c	5'-CCTGGATAGCCTTTCTTC-3'	5'-CGTCCATGTGAAAATGTG-3'	5'-FAM-TTCTTGTCTTGGCTTCAACTTGGA-TAMRA-3'
Mrc1	5'-AGGAGTTCATTATACAAACTG-3'	5'-CACCAATCACAACTACAC-3'	5'-FAM-TAGGCTACTTCTTCTTCCACCAGG-TAMRA-3'
IL-10	5'-GCTGGACAACATACTGCTAACC-3'	5'-CCTGGGGCATCACTTCTACC-3'	5'-FAM-TGGCAACCCAAGTAACCCTTAAAGTCCTGC-TAMRA-3'

Supplemental Table S2. Primary antibodies used in this study

Antibody Species	Antigen	Method	Company
goat polyclonal	mouse MRP8	IHC, WB	R&D systems, Minneapolis, MN, USA
rat monoclonal	mouse Mac-2	IHC	Cedarlane, Burlington, Ontario, Canada
mouse monoclonal	mouse pIκB	WB	Cell Signaling, Boston, MA, USA
rabbit polyclonal	NFkB p65 subunit	WB	Cell Signaling, Boston, MA, USA
mouse monoclonal	β-actin	WB	Sigma Aldrich, St Louis, MO, USA
mouse monoclonal	mouse GAPDH	WB	Santa Cruz Biotechnology, Santa Cruz, CA, USA
rat monoclonal	mouse CD16/32(FcγⅢ/Ⅱ)	FCM	BD biosciences, Bedford, MA, USA
rat monoclonal	mouse CD11b	FCM	BD biosciences, Bedford, MA, USA
rat monoclonal	mouse CD54(ICAM-1)	FCM	Invitrogen, San Diego, CA, USA
rat monoclonal	mouse L-selectin(CD62L)	FCM	Biolegend, San Diego, CA, USA
rat monoclonal	mouse Mincle	FCM	MBL, Nagoya, Aichi, Japan

Supplemental Figure S1
Generation of a conditional MRP8 gene.

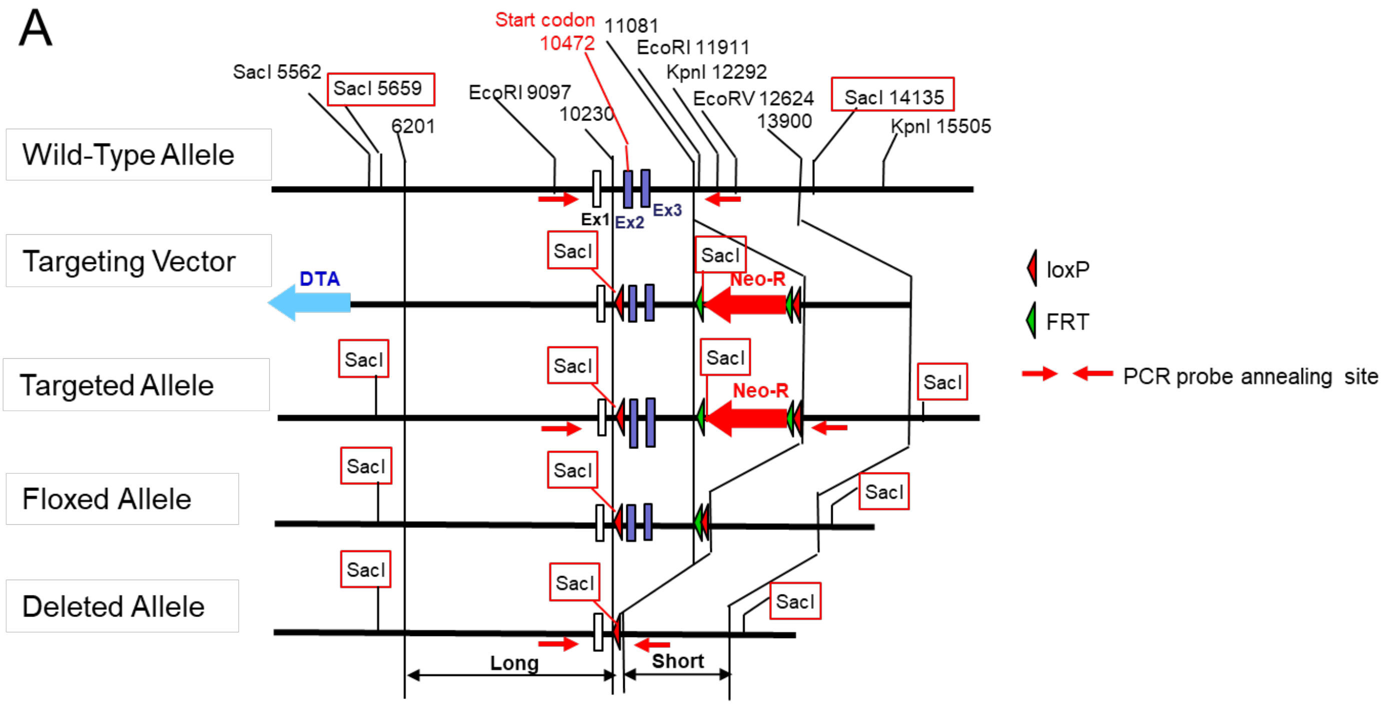

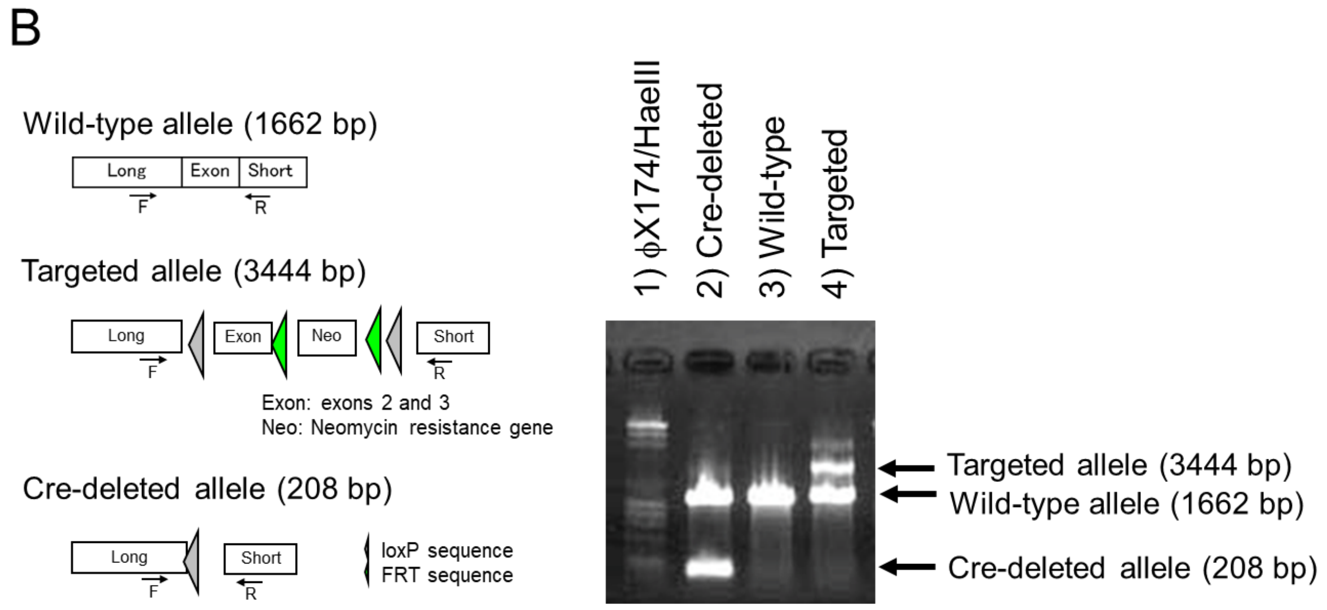

Supplemental Figure S2
Evaluation of recombination efficiency in MyM8KO mice.

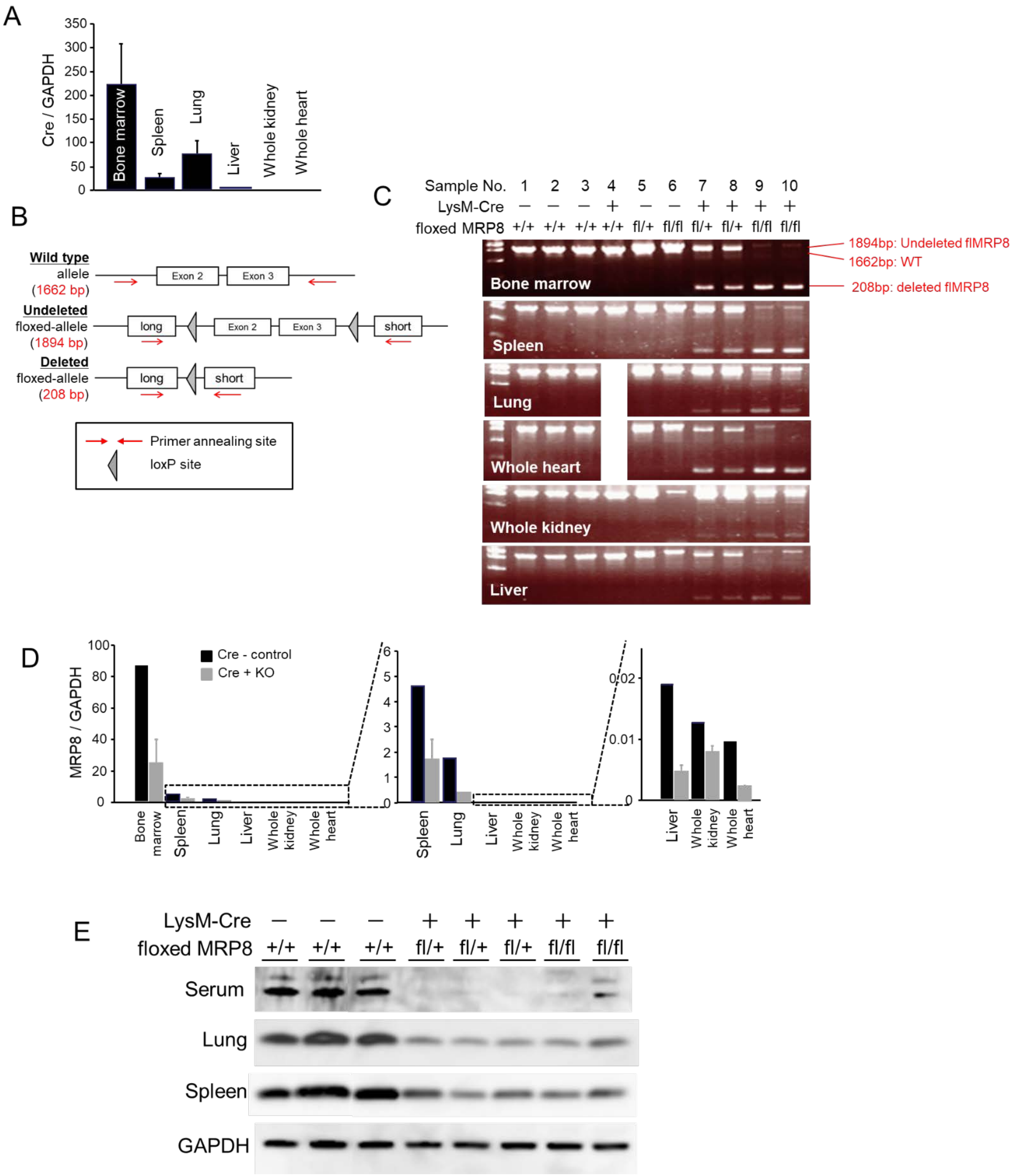

Supplemental Figure S3
MRP8 positivity of Mac2-positive macrophages in glomeruli and tubulointerstitium in diabetic-hyperlipidemic mice, and localization of MRP8.

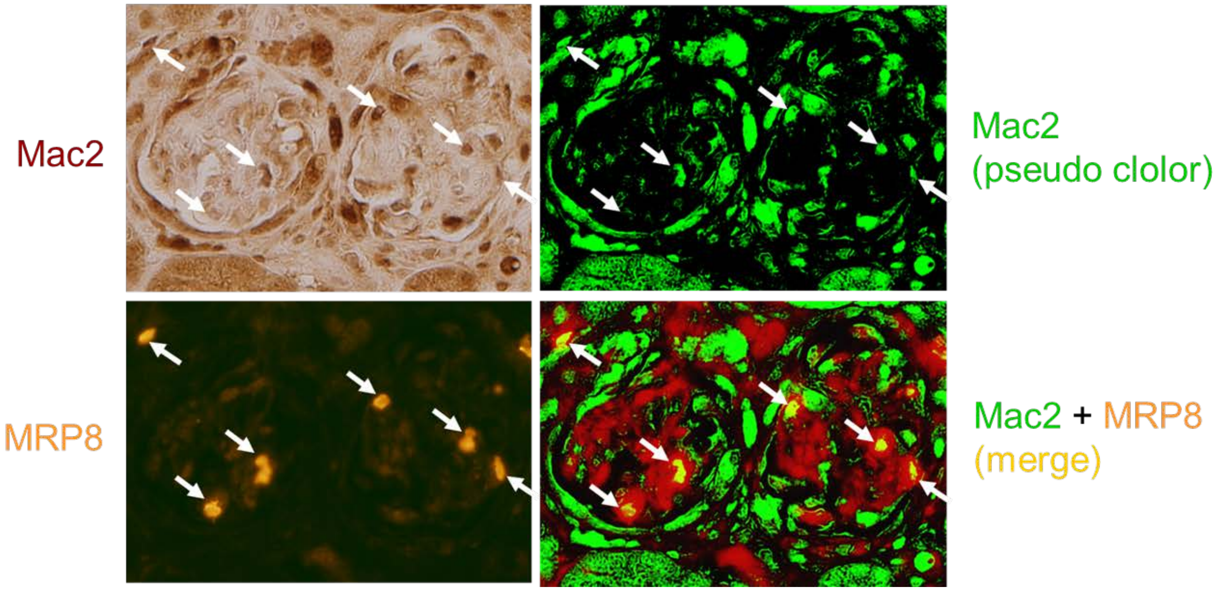

Supplemental Figure S4
Effects of stimulation with cultured medium of renal intrinsic cells on TLR4 and MRP8 expressions in RAW264.7 mouse Mϕ.

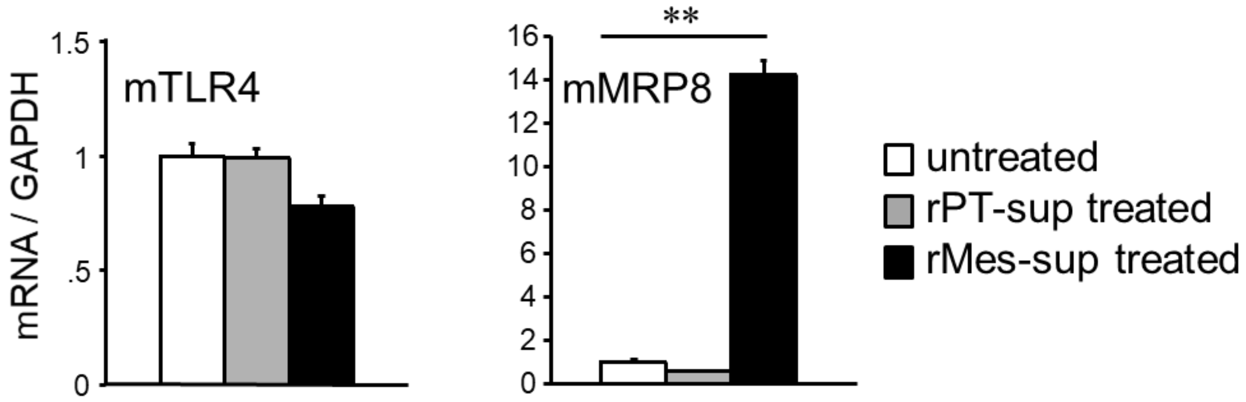

Supplemental Figure S5

Effects of E5564, a TLR4 antagonist, on Mϕ treated with mesangial cell-cultured medium.

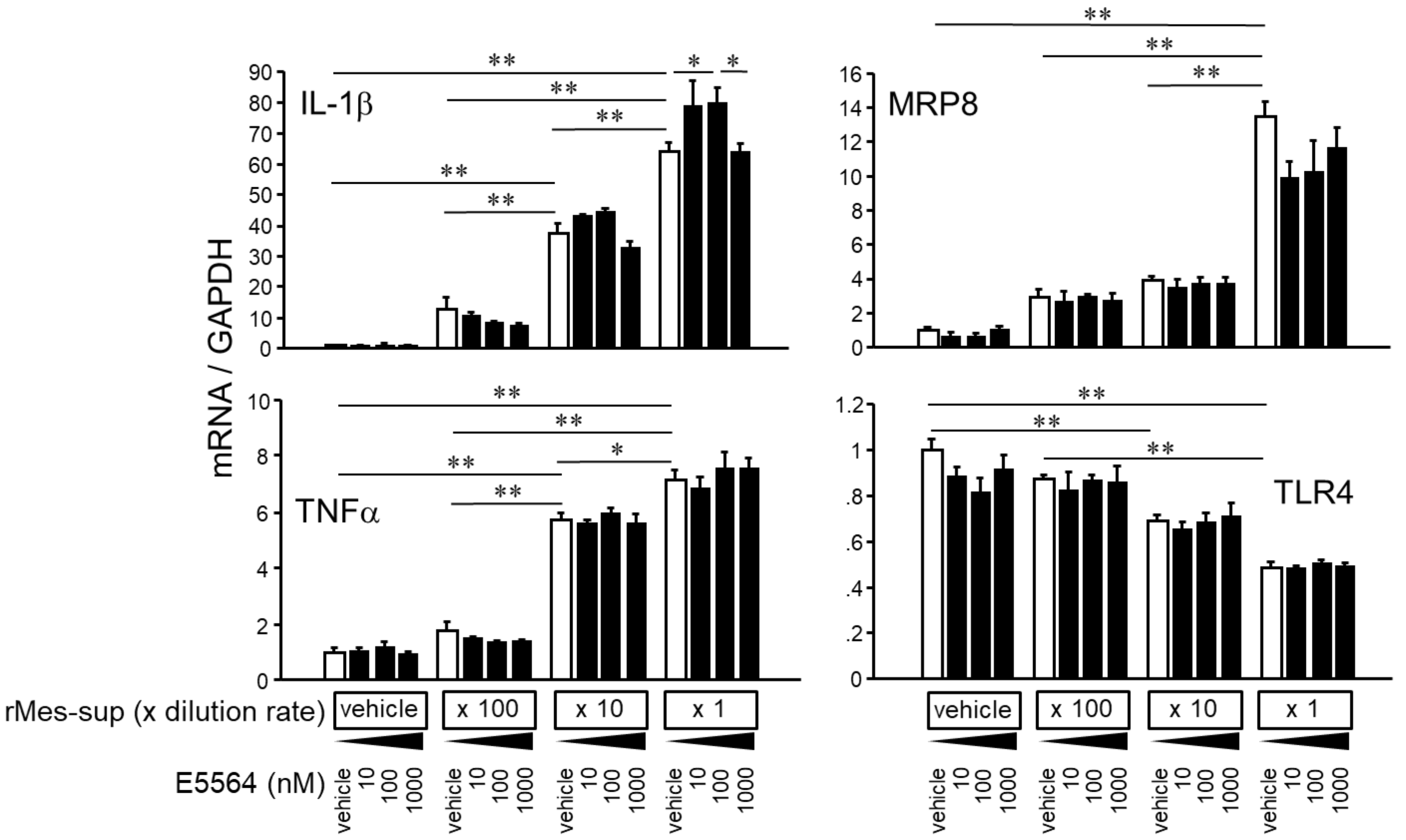

Supplemental Figure S6

Effects of E5564, a TLR4 antagonist, on Mϕ treated with proximal tubular cell-cultured medium.

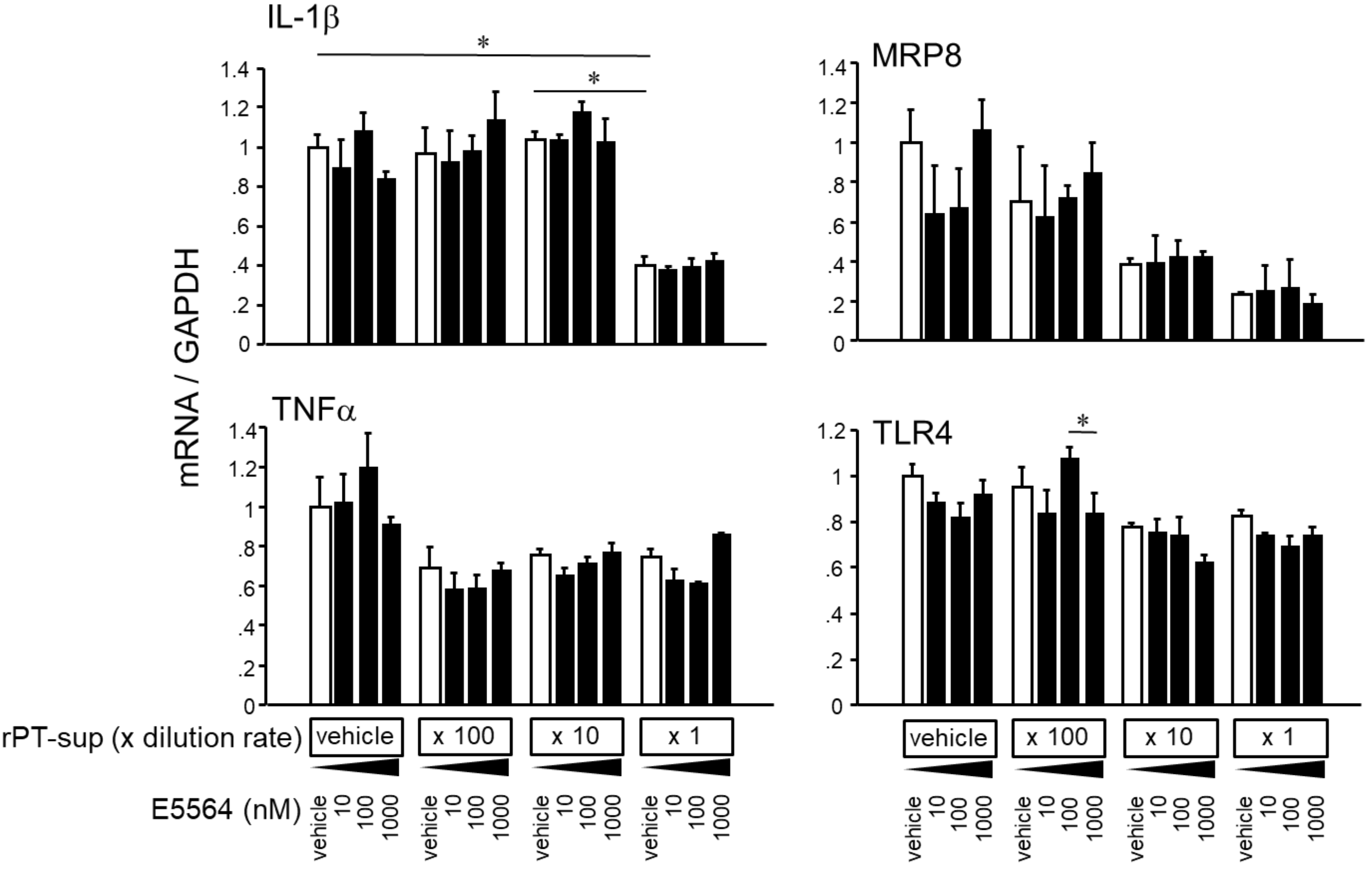

Supplemental Figure S7

Effects of MRP8 deletion on LPS-induced pro-inflammatory phenotypic changes in Mϕ.

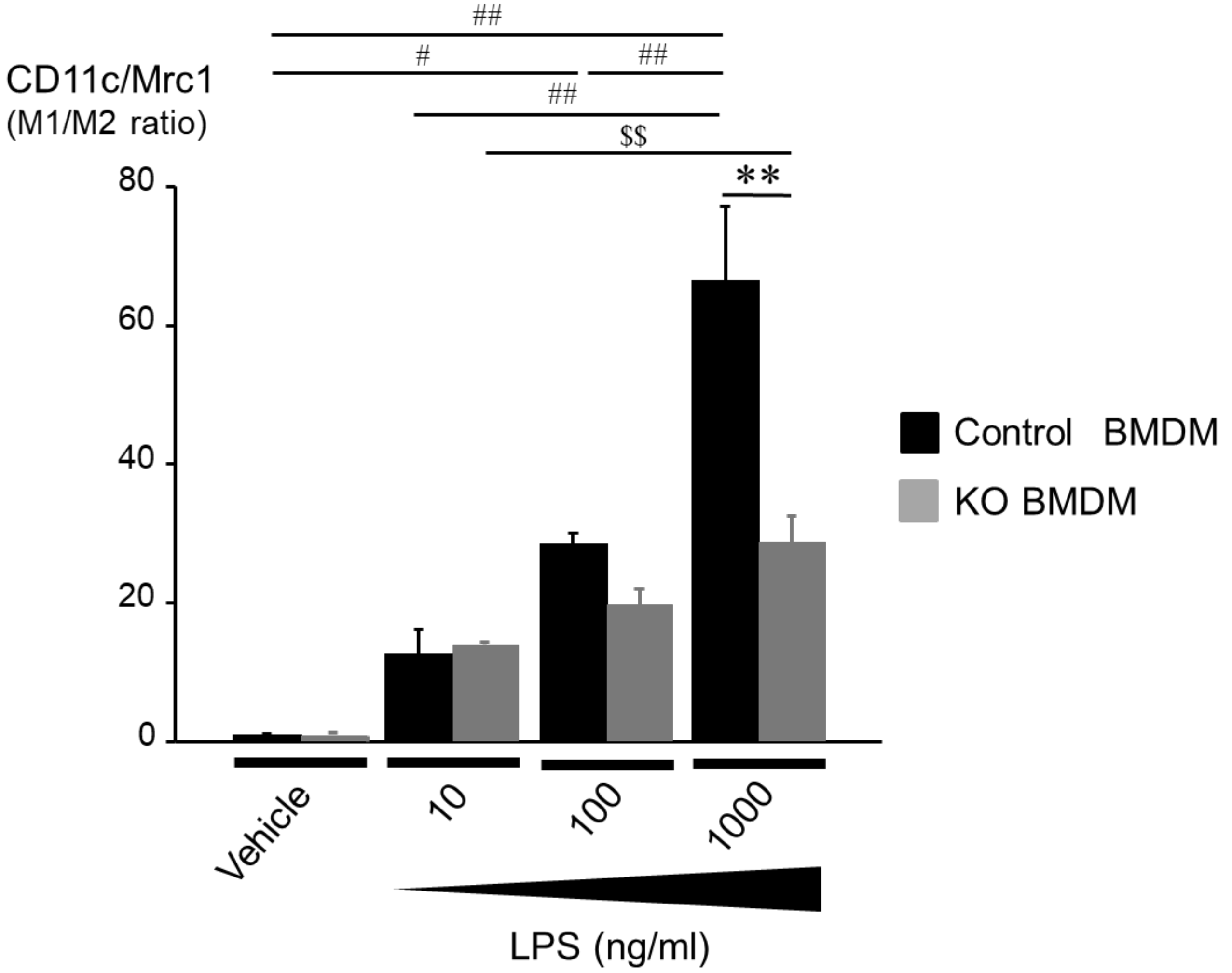

Supplemental Figure S8

Effects of myeloid lineage cell-specific deletion of MRP8 on mRNA expressions by TaqMan real-time RT-PCR in the whole kidney and isolated glomeruli.

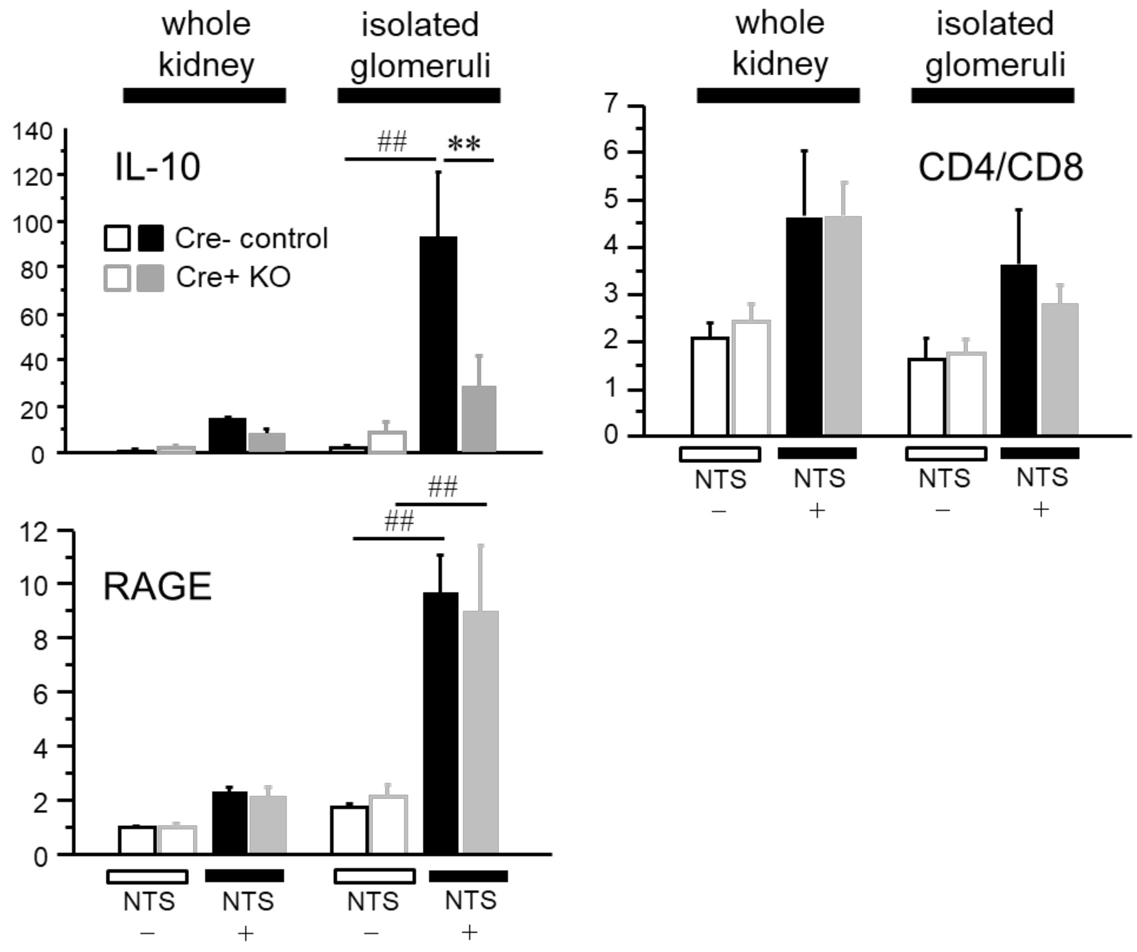

Supplemental Figure S9
Evaluation of stress fiber formation in BMDM by phalloidin staining.

A

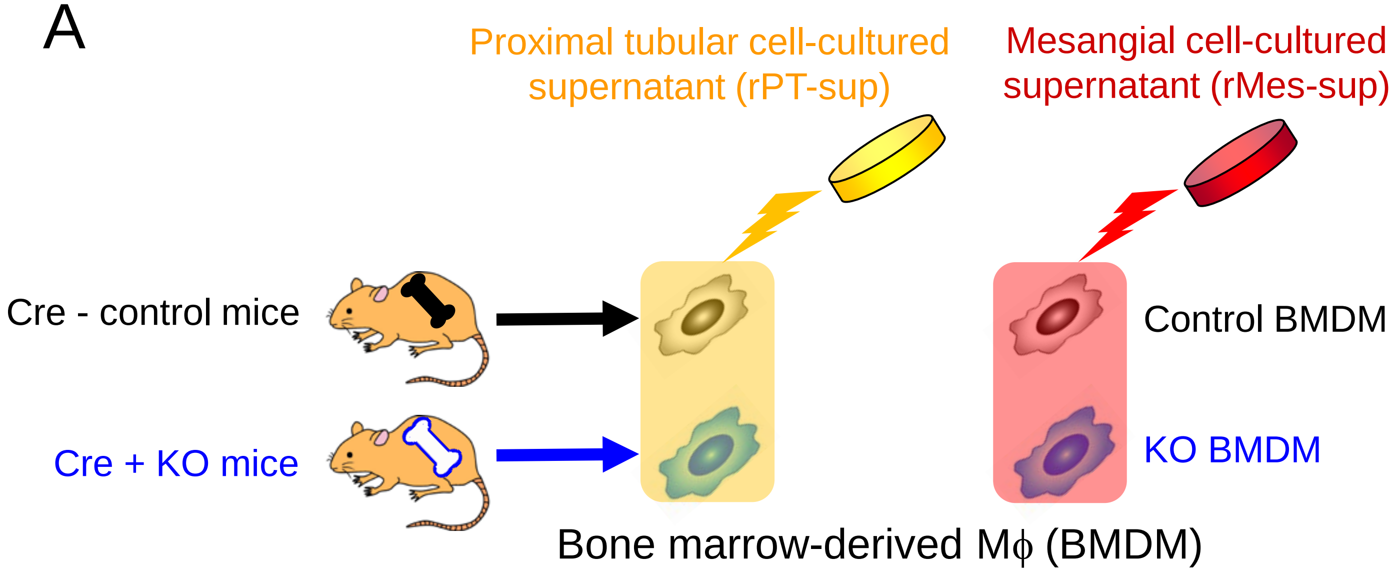

B

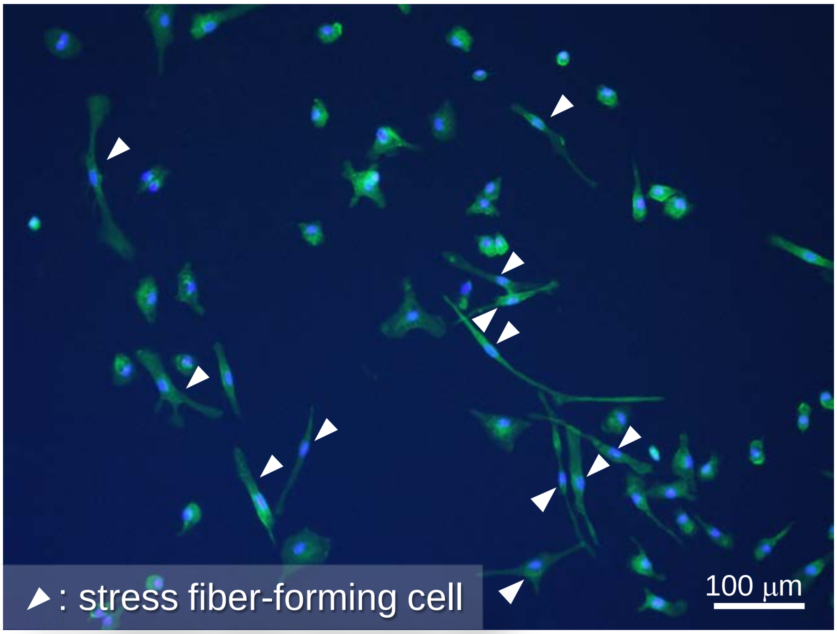

Supplemental Figure S10
Flow cytometry (FCM) of peripheral blood cells in MyM8KO non-NTN, healthy mice.

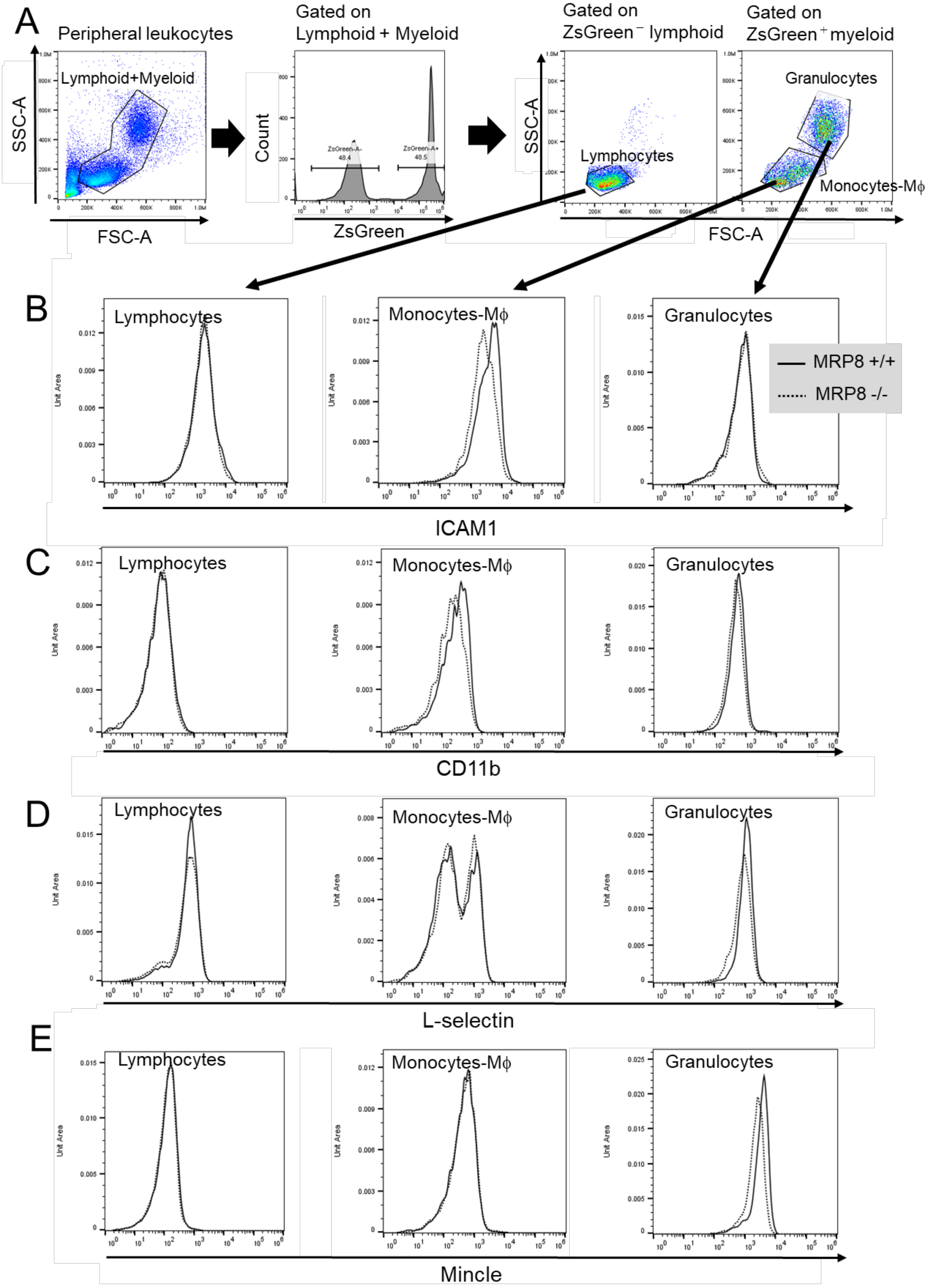

Supplement: Supplementary file 1 — Supplementary information. [file 41598_2020_59970_MOESM1_ESM.pdf]
